# Aerobic Exercise Inhibited P2X7 Purinergic Receptors to Improve Cardiac Remodeling in Mice With Type 2 Diabetes

**DOI:** 10.3389/fphys.2022.828020

**Published:** 2022-05-31

**Authors:** Ting Wang, Jianmin Li, Hui Li, Xin Zhong, Luya Wang, Shujue Zhao, Xuesheng Liu, Zhouqing Huang, Yonghua Wang

**Affiliations:** ^1^ The Key Laboratory of Cardiovascular Disease of Wenzhou, Department of Cardiology, The First Affiliated Hospital of Wenzhou Medical University, Wenzhou, China; ^2^ Department of Pathology, The First Affiliated Hospital of Wenzhou Medical University, Wenzhou, China; ^3^ Department of Ultrasound, The First Affiliated Hospital of Wenzhou Medical University, Wenzhou, China; ^4^ Department of Physical Education, Wenzhou Medical University, Wenzhou, China

**Keywords:** aerobic exercise, P2X7 purinergic receptors, diabetic cardiomyopathy, cardiac remodeling, fibrosis, apoptosis

## Abstract

**Background:** Diabetic cardiomyopathy (DCM), the main complication of diabetes mellitus, presents as cardiac dysfunction by ventricular remodeling. In addition, the inhibition of P2X7 purinergic receptors (P2X7R) alleviates cardiac fibrosis and apoptosis in Type 1 diabetes. However, whether exercise training improves cardiac remodeling by regulating P2X7R remains unknown.

**Methods:** Db/db mice spontaneously induced with type 2 diabetes and high-fat diet (HFD) and mice with streptozotocin (STZ)-induced type 2 diabetes mice were treated by 12-week treadmill training. Cardiac functions were observed by two-dimensional echocardiography. Hematoxylin-eosin staining, Sirius red staining and transmission electron microscopy were respectively used to detect cardiac morphology, fibrosis and mitochondria. In addition, real-time polymerase chain reaction and Western Blot were used to detect mRNA and protein levels.

**Results:** Studying the hearts of db/db mice and STZ-induced mice, we found that collagen deposition and the number of disordered cells significantly increased compared with the control group. However, exercise markedly reversed these changes, and the same tendency was observed in the expression of MMP9, COL-I, and TGF-β, which indicated cardiac fibrotic and hypertrophic markers, including ANP and MyHC expression. In addition, the increased Caspase-3 level and the ratio of Bax/Bcl2 were reduced by exercise training, and similar results were observed in the TUNEL test. Notably, the expression of P2X7R was greatly upregulated in the hearts of db/db mice and HFD + STZ-induced DM mice and downregulated by aerobic exercise. Moreover, we indicated that P2X7R knock out significantly reduced the collagen deposition and disordered cells in the DM group. Furthermore, the apoptosis levels and TUNEL analysis were greatly inhibited by exercise or in the P2X7R^−/−^ group in DM. We found significant differences between the P2X7R^−/−^ + DM + EX group and DM + EX group in myocardial tissue apoptosis and fibrosis, in which the former is significantly milder. Moreover, compared with the P2X7R^−/−^ + DM group, the P2X7R^−/−^ + DM + EX group represented a lower level of cardiac fibrosis. The expression levels of TGF-β at the protein level and TGF-β and ANP at the genetic level were evidently decreased in the P2X7R^−/−^ + DM + EX group.

**Conclusion:** Aerobic exercise reversed cardiac remodeling in diabetic mice at least partly through inhibiting P2X7R expression in cardiomyocytes.

## Introduction

In recent years, the global prevalence of diabetes, particularly type 2 diabetes mellitus (T2DM), has been increasing in a disturbing manner (Guariguata et al., 2014; Cho et al., 2018; Ritchie and Abel, 2020). Diabetic cardiomyopathy (DCM) is a comorbidity of diabetes mellitus, characterized by impairment of the myocardial structure and functional damage in the absence of coronary atherosclerosis, valvular disease, and overt clinical coronary artery disease (CAD) (Zheng et al., 2018; Zhang and Hao, 2020). In addition, DCM is manifested in cardiac enlargement, hypertrophy, myocardial lipid accumulation, fibrosis, and cardiac dysfunction (Liu et al., 2015). For the underlying mechanism of DCM, cells oxidation, excessive production of reactive oxygen species (ROS), and endoplasmic reticulum stress are thought to be drivers of cell death, including apoptosis and autophagy, which contribute to subsequent replacement fibrosis, followed by deterioration of heart function (Chowdhry et al., 2007; Mellor et al., 2011; Bugger and Abel, 2014; Gibb and Hill, 2018).

P2X7R, a neuronal P2X receptor, is an ATP-gated cationic channel composed of three subunits (Miras-Portugal et al., 2017). Considerable evidence has shown that P2X7R is involved in the regulation of multiple disease progression. For example, the inhibition of P2X7R can prevent pancreatic fibrosis in mice with chronic pancreatitis (Zhang et al., 2017), lead to the reduction of inflammation (Solini and Novak, 2019; Wu et al., 2020), hepatocyte apoptosis (Huang et al., 2014; Baeza-Raja et al., 2020), and ureteral obstructive reaction collagen deposition (Gonçalves et al., 2006). In recent years, the role of P2X7R in cardiovascular disease has received considerable attention (Chen et al., 2018; Ding et al., 2019), with the expression of P2X7R in coronary artery and myocardial tissue becoming a consensus. Studies have revealed that P2X7R is involved in the progression of atherosclerosis (Peng et al., 2015; Zhou et al., 2021). Furthermore, P2X7R inhibitors can reduce ischemia-reperfusion damage in animal models (Granado et al., 2015), improve myocardial ischemia injury (Tu et al., 2013), and reduce cardiac fibrosis induced by TGFβ through regulating the NLRP3/IL-1beta pathway (Zhou et al., 2020). Additionally, our previous report demonstrates that it’s important for P2X7R to regulate cardiac fibrosis in type 1 diabetes (Huang et al., 2021). However, the potential role of P2X7R on type 2 diabetes remains unclear.

The benefits of exercise training have received widespread attention. For instance, exercise has been reported to elicit a large influx of immune cells in tumors and reduced tumor incidence and growth by more than 60% in multiple mouse models (Idorn and Thor Straten, 2017). Regular exercise reduces hypersensitivity in rodent models of chronic pain (Pitcher, 2018), prevents and treats stage 1–2 hypertension in postmenopausal women (Lin and Lee, 2018), and manages the common effects of Multiple Sclerosis, Stroke, and Parkinson Disease (Kim et al., 2019). In cardiovascular terms, exercise is regarded as a diagnostic and prognostic tool for chronic heart failure and an important measure for therapeutic intervention (Cattadori et al., 2018). In addition, exercise is an efficacious approach for treating diabetes complications (Yaribeygi et al., 2019). Based on previous reports, exercise can improve DCM in mice by reducing ROS production, improving mitochondrial dysfunction and maintaining energy balance (Mahmoud, 2017; Wang et al., 2020), and in diabetic rats, exercise can reduce myocardial fibrosis and improve cardiac function by inhibiting the TGF-β1/Smad signaling pathway (Wang et al., 2019), enhancing the expression of miR-486a-5p and inhibiting myocardial cell apoptosis (Sun et al., 2021). However, whether the underling mechanism of exercise is associated with the regulation of P2X7R levels in mice with type 2 diabetes remains elusive. Thus, this study aimed to investigate the effect of aerobic exercise on P2X7R expression and followed-by cardiac remodeling regulating in diabetic mice.

## Materials and Methods

### Reagents

BCL-2 (ab196495) and GAPDH (#2881S) antibodies were obtained from Abcam (Cambridge, United Kingdom), whereas Caspase-3 (#9662S) antibody was purchased from Cell Signaling Technology (Danvers, MA, United States). TGF-β (A18692) antibody was purchased from Abclonal (Wuhan, China), and Bax (ET1603-34), MMP9 (ER1706-40), MyHC (ET1702-88), and P2X7R (ER1901-99) antibodies were obtained from Huabio (Hangzhou, China). The above antibodies were diluted at 1:1,000. In addition, Collagen 1 (sc293182) and ANP (sc-515701), which were diluted at 1:200, were obtained from Santa Cruz Biotechnology (Dallas, TX, United States). Goat anti-rabbit secondary antibodies (A0208) and goat anti-mouse secondary antibodies (A0216) which were used in the Western blot and One-Step TUNEL Apoptosis Assay Kit, respectively, were purchased from Beyotime (Shanghai, China). Hematoxylin-eosin (H&E) Staining Kit and Masson’s Trichrome Stain Kit were obtained from Solarbio (Beijing, China). STZ was purchased from Sigma (CA, United States) and citric acid-sodium citrate buffer was purchased from Solarbio (Beijing, China).

### Animals

This study was conducted out in accordance with the principles of the Guide for the Care and Use of Laboratory Animals (National Research Council, United States). The protocol was approved by the Institutional Animal Care and Use Committee, Wenzhou Medical University (wydw2016-0266). Six-week-old male lean control (db/+) and diabetic obese (db/db) mice with C57BL/6J background were purchased from Model Animal Research Center of Nanjing University. Purinergic P2X7 receptor knockout (P2X7R^−/−^) mice with C57BL/6J background were purchased from Nanjing Institute of Biological Sciences. All mice were housed in specific pathogen-free conditions. The animals were kept under a 12 h/12 h light-dark cycle, and they were allowed free access to food and water.

### Experimental Exercise Protocol and Blood Sample Collection

After acclimatization for 1 week, P2X7R^−/−^ mice or wild-type mice were fed either a control diet (*n* = 24) or a high-fat diet (HFD; HD001; Medicine, Shenzhen, China; *n* = 32) for 4 weeks. Next, the diabetic mice were injected with 25 mg/(kg d) of streptomycin for five consecutive days, and they were classified as diabetic mice after observation of fasting blood glucose ≥11.1 mmol/L for two consecutive days after 1 week. Then, HFD was continued. After 12 weeks, diabetic mice were randomly divided into two groups: sedentary mice without exercise training (DM, *n* = 6–8) and mice with regular aerobic exercise training for 12 weeks (DM + EX, *n* = 8). Notably, sedentary mice were fed a standard diet, and they served as the control group (WT, *n* = 8). The sedentary diabetic obese (db/db) mice were fed a standard diet, and they were divided into two groups: sedentary mice (db/db, *n* = 10) and regular aerobic exercise trained mice (db/db + EX, *n* = 10). The exercise experiment was conducted using a small animal treadmill (#1050 RM-E57) from Columbus instruments with zero inclination. Mice in the exercise groups were trained on a motor treadmill at 5 m/min for 60 min on the first day. Initial adaptation was performed at 7 m/min for 5 days. The running speed was then increased by 1 m/min each day until the speed reached 10 m/min at the end of the training protocol (Fernando et al., 1993). Afterward, the mice were monitored to cover a daily distance at 10 m/min for the next 12 weeks, and they were trained for 5 days/week. Notably, all training sessions were performed during the afternoon (2:00–5:00 p.m.). After 12 weeks of treadmill exercise, mice were starved for 12 h, and then they were anesthetized with an intraperitoneal injection of pentobarbital sodium (50 mg/kg). Blood samples were collected from the inferior vena cava into EDTA tubes. The plasma was immediately separated by centrifugation at 3,000 rpm for 10 min and stored at 80°C until chemical assay analysis.

### Detection of Cardiac Function in Mice

Cardiac systolic and diastolic functions were measured using two-dimensional echocardiography. After anesthesia, the hair in the precardiac area was removed, and then the ultrasonic probe was used for ultrasonic detection. Acuson-sequoia 512 was used and equipped with an acuson-15L8w probe at 12–14 MHz. Images were acquired in the M-mode and short-axis, and waveforms and related data were recorded. Ejection fraction (EF) was calculated using the following equation: EF = [(LVEDV − LVESV)/LVEDV] * 100%. Moreover, fractional shortening (FS) was calculated using the following equation: FS = [(LVIDd − LVIDs)/LVIDd] * 100%.

### Real-Time Polymerase Chain Reaction Analysis

Total RNA was extracted from mice hearts using TRIzol Reagent following the manufacturer’s protocol (Invitrogen Life Technologies). One microgram of total RNA from each sample was used to generate cDNAs using the RevertAid TM First Strand cDNA Synthesis Kit (#K1622; Thermo) following the manufacturer’s instructions. The resultant cDNA was amplified using SYBR Green Qpcr Super Mix-UDG kit (#RR037A; Takara). In addition, the PCR reaction was directly monitored by the CFX96 Touch TM Real-Time PCR detection system. All results were normalized against GAPDH (B661204; Sangon Biotech, Shanghai, China).

Real-time PCR was conducted using the following primers:

P2X7R: Forward primer: CCA​AGG​TCA​AAG​GCA​TAG​CAG​AGG
Reverse primer: TAG​GAC​ACC​AGG​CAG​AGA​CTT​CAC
MyHC: Forward primer: CAA​AGG​CAA​GGC​AAA​GAA​AG
Reverse primer: TCA​CCC​CTG​GAG​ACT​TTG​TC
MMP9: Forward primer: GCA​GAG​GCA​TAC​TTG​TAC​CG
Reverse primer: TGA​TGT​TAT​GAT​GGT​CCC​ACT​TG
Collagen I: Forward primer: GAG​GGC​GAG​TGC​TGT​GCT​TTC
Reverse primer: GGA​GAC​CAC​GAG​GAC​CAG​AAG​G
Bax: Forward primer: CCG​GCG​AAT​TGG​AGA​TGA​ACT
Reverse primer: CCA​GCC​CAT​GAT​GGT​TCT​GAT
Bcl-2: Forward primer: GCT​ACC​GTC​GTG​ACT​TCG​C
Reverse primer: CCC​CAC​CGA​ACT​CAA​AGA​AGG
Caspase-3: Forward primer: CTG​ACT​GGA​AAG​CCG​AAA​CTC
Reverse primer: CGA​CCC​GTC​CTT​TGA​ATT​TCT
ANP: Forward primer: AAG​AAC​CTG​CTA​GAC​CAC​CTG​GA
Reverse primer: TGC​TTC​CTC​AGT​CTG​CTC​ACT​CAG
TGF-β: Forward primer: ACC​GCA​ACA​ACG​CCA​TCT​ATG​AG
Reverse primer: AGC​CCT​GTA​TTC​CGT​CTC​CTT​GG
GAPDH: Forward primer: ACC​CAG​AAG​ACT​GTG​GAT​GG
Reverse primer: TTC​AGC​TCA​GGG​ATG​ACC​TT

### Western Blot Analysis

Heart tissue samples (50–100 mg) and cardiomyocyte samples were ground and centrifuged at 12,000 g for 15 min, and then the supernatants were collected. The protein samples were separated by SDS-PAGE gel and transferred to a PVDF membrane (MERCK, Germany). Membranes were blocked with a 5% fat-free milk solution for 1 h at room temperature and subsequently incubated overnight with the primary antibodies at 4°C. After washing three times, immunoreactive bands were incubated with horseradish peroxidase-conjugated secondary antibody at room temperature for 1 h. Finally, proteins were detected *via* enhanced chemiluminescence (Bio-Rad, United States).

### Terminal Deoxynucleotidyl Transferase-Mediated DUTP Nick End Labeling Staining

The terminal deoxynucleotidyl transferase-mediated DUTP nick end labeling (TUNEL) assay was performed following the manufacturer’s instructions of One-Step TUNEL Apoptosis Assay Kit (Beyotime, Shanghai, China). TUNEL positive cells were imaged under a fluorescence microscope (400× amplification; Nikon, Japan).

### Hematoxylin and Eosin Staining, Scanning Electron Microscopy, Sirius Red Staining, and Immunohistochemistry Examination

Fresh tissues were fixed in 4% paraformaldehyde and embedded in paraffin. Then, the tissues were cut into 5 μm sections, followed by deparaffinization and rehydration as previously described. After rehydration, the sections were stained with H&E. Next, paraffin sections were stained using a Sirius Red Kit to evaluate the level of collagen deposition and fibrosis. The stained sections were then viewed under the Nikon microscope (Nikon, Japan). For immunohistochemical staining, tissue sections were deparaffinized with xylene, rehydrated in graded alcohol series, and subjected to antigen retrieval in 0.01 M of citrate buffer (pH 6.0) by microwaving. Next, the sections were placed in 3% hydrogen peroxide methanol for 30 min at room temperature. Slides were then blocked with 1% bovine serum albumin in phosphate-buffered saline for 30 min and incubated with primary antibody at 4°C overnight (P2X7R, 1:1,000). Afterward, slides were incubated with peroxidase-conjugated secondary antibodies (Santa Cruz, Dallas, TX, United States, 1:1,000 dilution) for 1 h at room temperature. Finally, slides were counterstained with hematoxylin for 5 min, dehydrated, and mounted. Each image of the sections was captured using a light microscope (×400 amplification; Nikon, Shinagawa, Tokyo, Japan). Notably, samples were collected on the basis of the following key points: small, fast, cold, and accurate. After rinsing, 2.5% glutaraldehyde was fixed for 2 days. After fully rinsing, 1% osmium acid was fixed for 1.5 h, and then uranium acetate block was dyed, dehydrated and soaked. Finally, the samples were sectioned using an ultrathin slicer (RMC-PXL), and then the sections were embedded. Images were viewed and acquired by using a transmission electron microscope (H-7500).

### Statistical Analysis

All statistical analyses were performed using GraphPad Prism 8.0 software (GraphPad, San Diego, CA, United States), and all values were presented as the mean ± standard error of the mean. Multiple comparisons were analyzed by two-way ANOVA followed by the Tukey’s correction. For all tests, a *p*-value < 0.05 was considered significant.

## Results

### Aerobic Exercise Reduced the Expression of P2X7R in Cardiac Tissues of DM Mice

**FIGURE 1:**
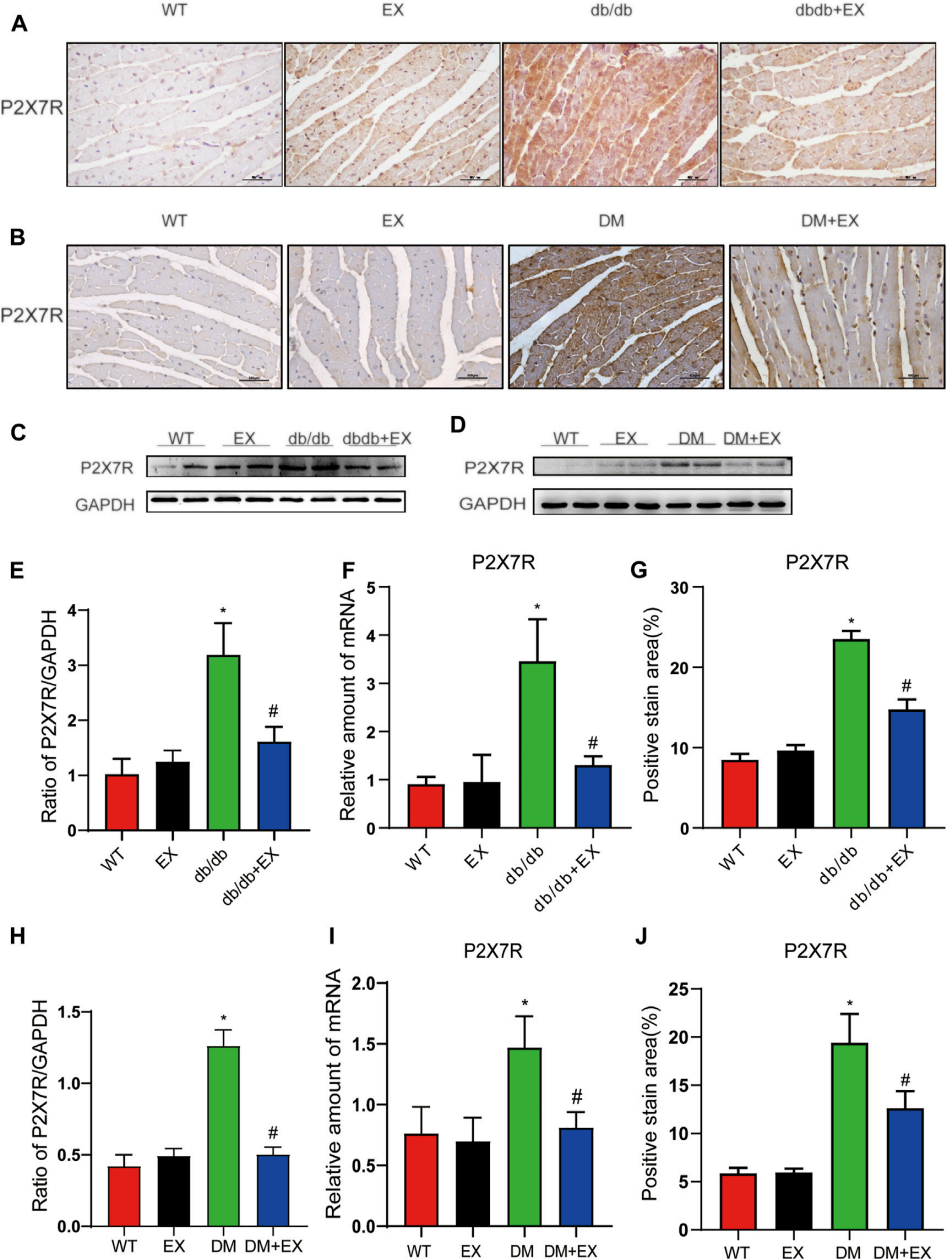
Regular aerobic exercise reduced the P2X7R levels in db/db mice or HFD + STZ-induced T2DM mice. **(A,B)** Immunohistochemistry of P2X7R. **(C,D)** Immunohistochemical analysis of P2X7R. **(E,F)** Representative Western blot analysis of P2X7R. **(G–J)** semi-quantification of protein and mRNA level of P2X7R. *p < 0.05 vs. the WT group, ^#^ *p* < 0.05 versus the db/db group or DM group. WT: the control group.

As shown in Figure 1, P2X7R expression was significantly increased in the heart of db/db mice or HFD + STZ-induced T2DM mice compared with control mice (Figures 1A–D) and decreased by 12-week aerobic exercise training. Consequently, we observed the same tendency of P2X7R level from the protein (Figures 1E–H) and gene levels (Figures 1I,J) in the DM group or DM + EX group, which was consistent with immunohistochemical staining. Collectively, these results showed that the P2X7 receptor was upregulated in the cardiac tissues of type 2 diabetes model and downregulated by aerobic exercise.

### Changes in Body Weight, Lipid and Blood Glucose Levels of Mice

**FIGURE 2:**
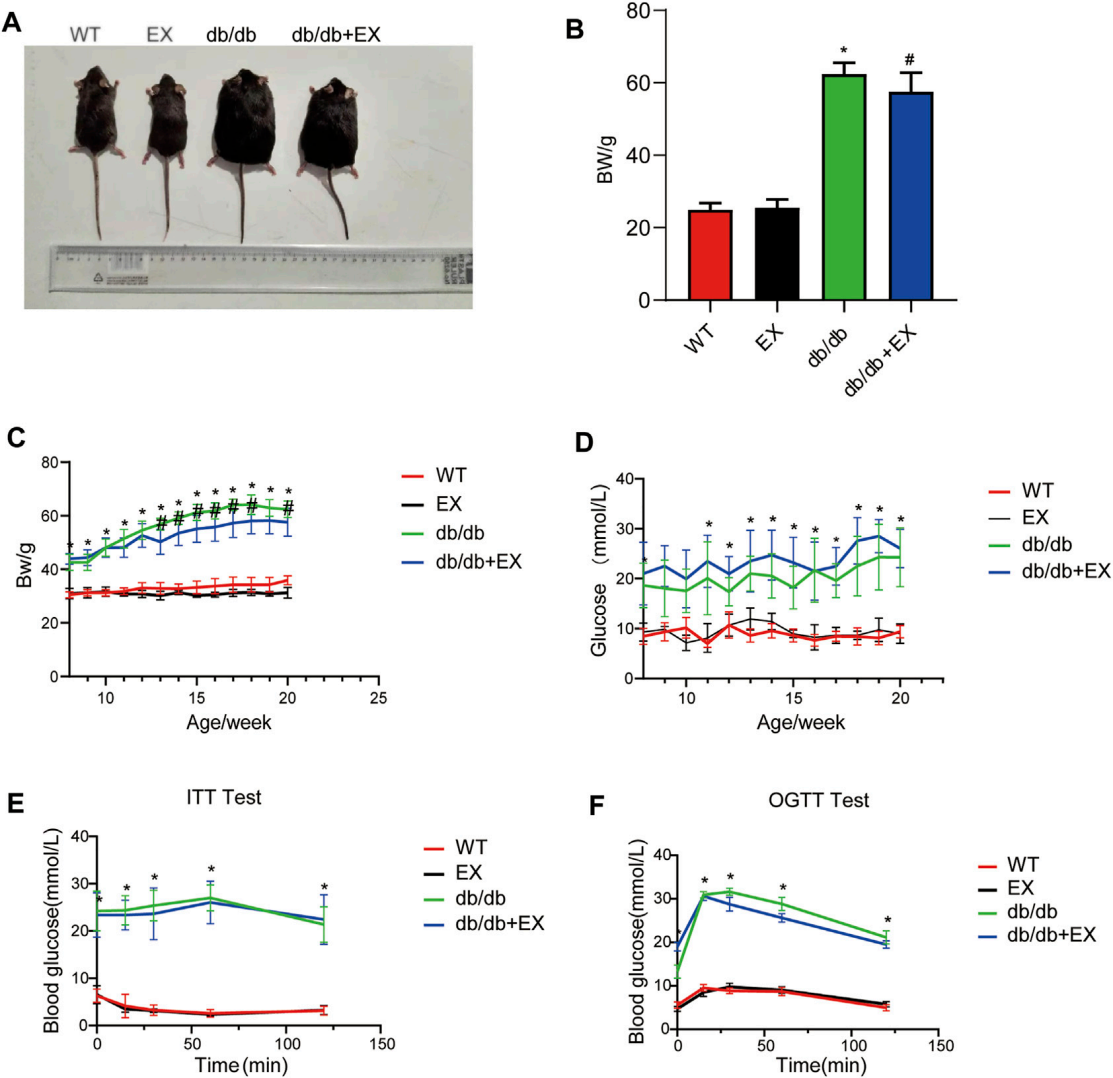
Changes in general characteristics of mice. **(A)** The appearance of mice after exercise training. **(B)** Weight of mice after exercise training. **(C–F)** Body weight and blood glucose level during exercise training in mice. *p < 0.05 vs. the WT group, ^#^ *p* < 0.05 versus the db/db group.

**FIGURE 3:**
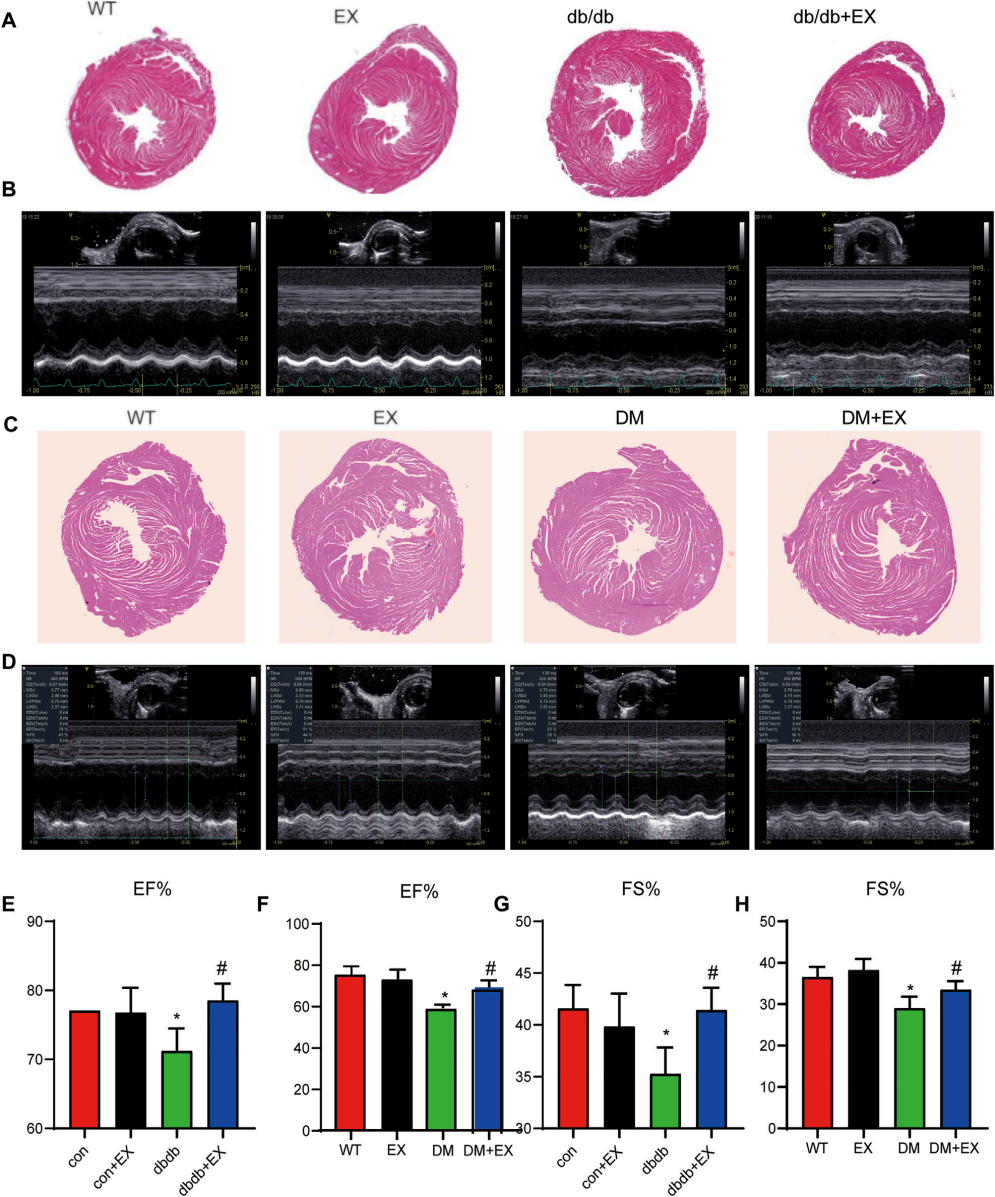
Exercise improved heart function in db/db mice or DM mice. **(A,C)** Cardiac morphology after exercise in db/db or DM mice. **(B,D)** M-mode echocardiography in mice. **(E–H)** Cardiac function in mice. *p < 0.05 vs. the WT group, ^#^ *p* < 0.05 vs. the DM group. EF%: ejection fraction; FS%: fraction shortening.

Various physiological parameters were measured to determine the impact of the moderate exercise regimen (Figure 2; Table 1). We regularly monitored the weight of mice and found that the weight of db/db mice greatly increased compared with WT and reduced by exercise (Figures 2A–C). In addition, the levels of total cholesterol (CHOL) and triglycerides (TRIG) were upregulated in the db/db group and downregulated in the exercise group (*p* < 0.05), whereas no significant changes in blood glucose levels were observed between the db/db and exercise groups (Figure 2D). Moreover, similar glucose level results can be observed in the model of HFD + STZ-induced mice (Figure 3E).

**TABLE 1:** Effects of exercise on the biochemistry of diabetic mice (±SD, *n* = 10).

	CHOL (mmol/L)	TRIG (mmol/L)	CK(U/L)
Normal range	0.93–2.48	0.62–1.63	68–1,070
WT	1.69 ± 0.33	0.88 ± 0.26	732.7 ± 181.7
EX	1.32 ± 0.38	0.93 ± 0.33	754.3 ± 297.2
db/db	4.02 ± 0.52^*^	2.00 ± 0.48^*^	641.5 ± 151.6
db/db + EX	3.16 ± 0.42^#^	1.15 ± 0.25^#^	950.5 ± 346.3

Data were presented as mean ± SD; TRIG, triglyceride; CHOL, cholesterol; CK, creatine kinase. ^*^ *p* < 0.05 vs. the WT group, ^#^ *p* < 0.05 vs. the db/db group.

### Aerobic Exercise Relieved Cardiac Dysfunction Induced by Type 2 Diabetes Mellitus Mice

Noninvasive transthoracic echocardiography was performed to examine the cardiac function of mice 2 h before sacrifice. The data from echocardiography showed that the heart rate was not affected. As shown in Figure 3B and Table 2, not only diastolic function disorder (as observed in IVSd and LVIDd) but also reduction of contraction function (EF%, FS%) was induced by diabetes. Consequently, these dysfunctions were substantially attenuated by aerobic exercise training in db/db mice, but no significant changes were observed between the WT group and EX group. Moreover, the consistent data trend was observed from the model of HFD + STZ-induced mice (Figures 3D,F,H). Figure 3D shows a representative echocardiogram of each group, which could be intuitively presented. Collectively, these results indicated that regular moderate intensity exercise can effectively improve cardiac function of diabetes mellitus.

**TABLE 2:** Cardiac function indexes of mice in each group (±SD, *n* = 10).

	WT	EX	db/db	db/db + EX
BW, g	24.90 ± 1.86	25.50 ± 2.24	62.34 ± 3.13*	56.51 ± 5.24^#^
HW, mg	141.4 ± 17.34	152.1 ± 17.7	162.5 ± 6.40	156.6 ± 58.28
HW/BW, mg/g	5.68 ± 0.51	5.96 ± 0.31	2.61 ± 0.16*	3.13 ± 0.23^#^
IVSd, mm	0.56 ± 0.06	0.58 ± 0.06	0.67 ± 0.08*	0.68 ± 0.06
LVIDd, mm	3.88 ± 0.26	3.88 ± 0.26	4.12 ± 0.22	4.12 ± 0.22
LVPWd, mm	0.58 ± 0.05	0.58 ± 0.05	0.70 ± 0.07*	0.63 ± 0.02^#^
LVIDs, mm	2.35 ± 0.21	2.38 ± 0.24	2.64 ± 0.18*	2.37 ± 0.12#
EF%	77.06 ± 3.90	76.79 ± 3.59	71.20 ± 3.28*	78.53 ± 2.46^#^
FS%	41.59 ± 2.27	39.84 ± 3.19	35.26 ± 2.57*	41.43 ± 2.17^#^

Echocardiographic assessment of cardiac function parameters of each group of experimental mice. HW/BW, heart weight/body weight; BW: weight; HW, heart weight; IVSd, diastolic interventricular septal thickness; LVIDd, diastolic left ventricle internal volume; LVPWd, left ventricular posterior wall thickness; LVIDs, left ventricular end-systolic diameter; EF%, ejection fraction; FS%, fraction shortening (*n* = 10; *p < 0.05 vs. the WT group, ^#^ *p* < 0.05 vs. the db/db group).

### Regular Aerobic Exercise Inhibited Myocardial Apoptosis in Type 2 Diabetes Mellitus Mice

**FIGURE 4:**
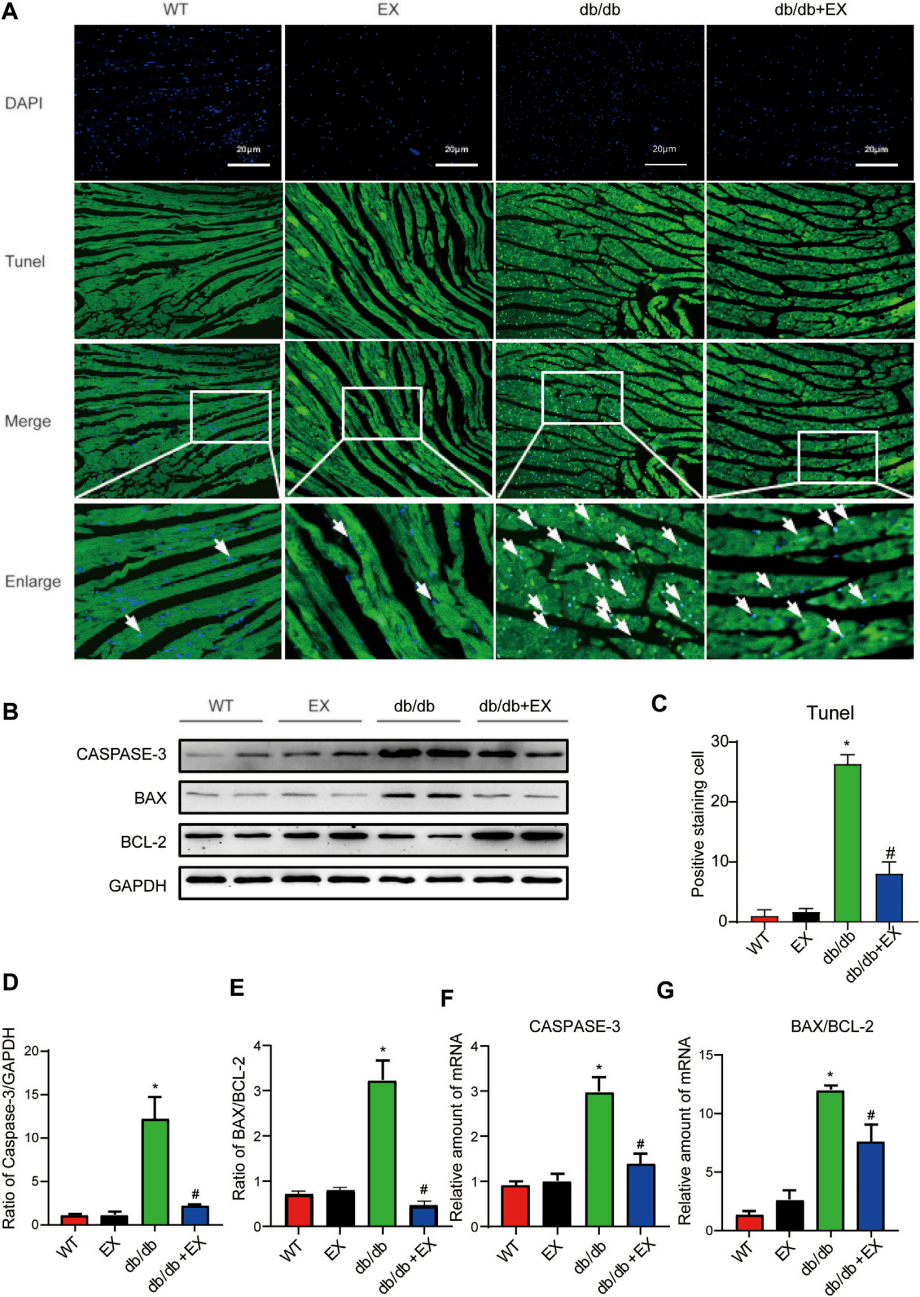
Exercise alleviated cardiomyocyte apoptosis in db/db mice. **(A)** TUNEL staining and quantitative analysis of myocardial tissue in mice **(C)**; **(B,D,E)** Western blot analysis was performed to detect apoptosis-related proteins (Caspase-3, Bcl-2, and Bax). **(F,G)** Gene expression of apoptotic protein. *p < 0.05 vs. the WT group, ^#^ *p* < 0.05 vs. the db/db group.

**FIGURE 5:**
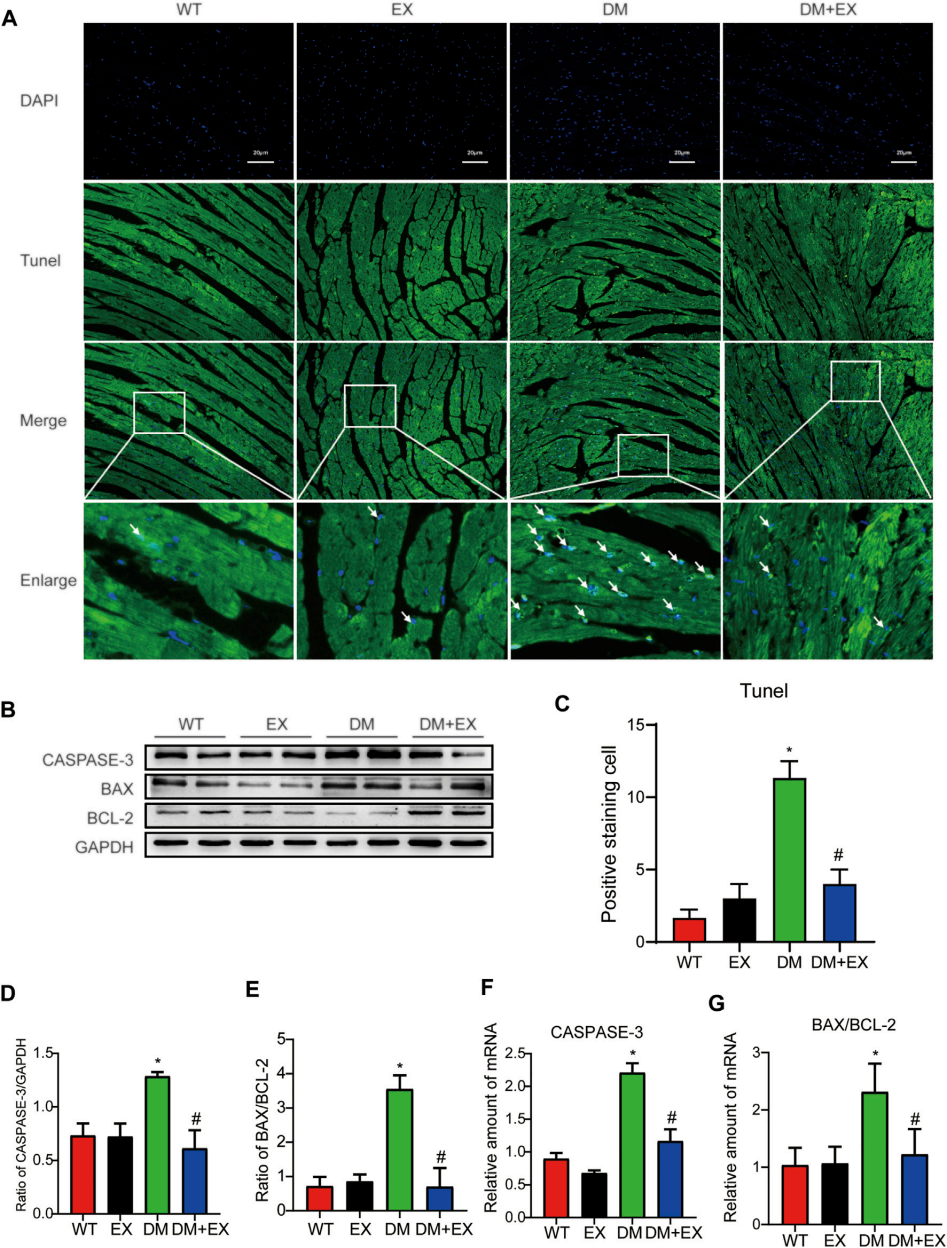
Exercise alleviated cardiomyocyte apoptosis in HFD + STZ-induced T2DM mice. **(A,C)** TUNEL staining and quantitative analysis of myocardial tissue in mice; **(B,D,E)** Western blot analysis was performed to detect apoptosis-related proteins (Caspase-3 and Bax) and anti-apoptotic protein (Bcl-2); **(F,G)** Gene expression of apoptosis-related proteins. *p < 0.05 vs. the WT group, ^#^ *p* < 0.05 vs. the DM group.

Notably, apoptosis plays a pivotal role in heart injury in DCM. As shown in cardiac TUNEL staining, more positive cells of apoptosis emerged in db/db mice than in the WT group, whereas less positive cells existed in db/db + EX mice than in db/db mice (Figures 4A,C). The expression levels of apoptosis-related proteins, such as Caspase-3 and Bax, were increased, and anti-apoptotic protein Bcl-2 was decreased in db/db mice. Consequently, regular exercise led to the expression of Caspase-3, and the ratio of Bax to Bcl-2 was reduced (Figures 4B,D,E). In addition, the mRNA level of Caspase-3, Bax, and Bcl-2 showed similar results (Figures 4F,G). We also investigated this phenomenon in HFD + STZ-induced DM mice (Figure 5) and found that the increase of cardiac apoptosis caused by diabetes was markedly inhibited by exercise. Thus, these results indicated that exercise can effectively alleviate the apoptosis of centrifuge cells in DCM.

### Regular Aerobic Exercise Ameliorated Myocardial Remodeling in Db/Db Mice

**FIGURE 6:**
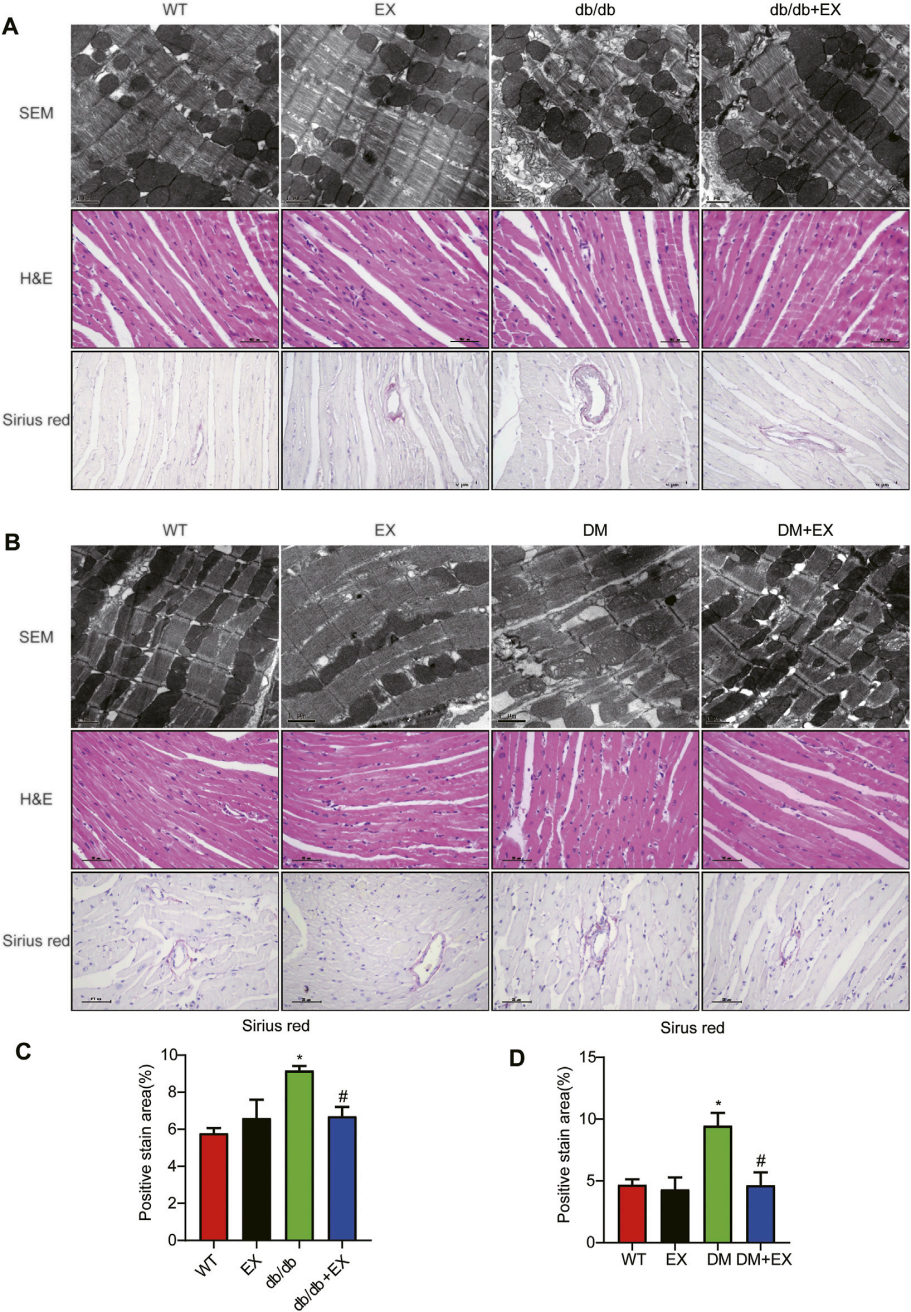
Exercise mitigated myocardial remodeling in diabetic mice. **(A,B)** SEM images of mouse myocardial tissue, hematoxylin–eosin (H&E) and Sirius red quantitative statistical images of Sirius red detecting myocardial fibrosis **(C,D)**. *p < 0.05 vs. the WT group, ^#^ *p* < 0.05 vs. the db/db group.

Given that aerobic exercise enhances the heart function of diabetic mice, we investigated whether it could improve cardiac remodeling. Thus, H&E staining and electron microscopy were performed to detect cardiac structure morphology. We observed significant structural abnormalities in cardiac tissues, such as disorganized myofibers caused by diabetes mellitus. In the WT group, the myocardial cells were rich in myofibrils, with clearly visible M and Z lines, round or elliptic mitochondria exhibiting many cristae and orderly arrangement, and myofilaments arranged tightly and neatly. By contrast, in cardiac tissues of the db/db group, the content of myofibrils was significantly reduced. The M and Z lines of cardiomyocytes were blurred, and the muscle filaments were broken and disordered. Moreover, we observed that mitochondrial disorder appeared as swelling deformation and cristae fracture with vacuolation in cardiac tissue of db/db mice. These disorder phenomena were markedly improved in the db/db + EX group (Figures 6A,B).

**FIGURE 7:**
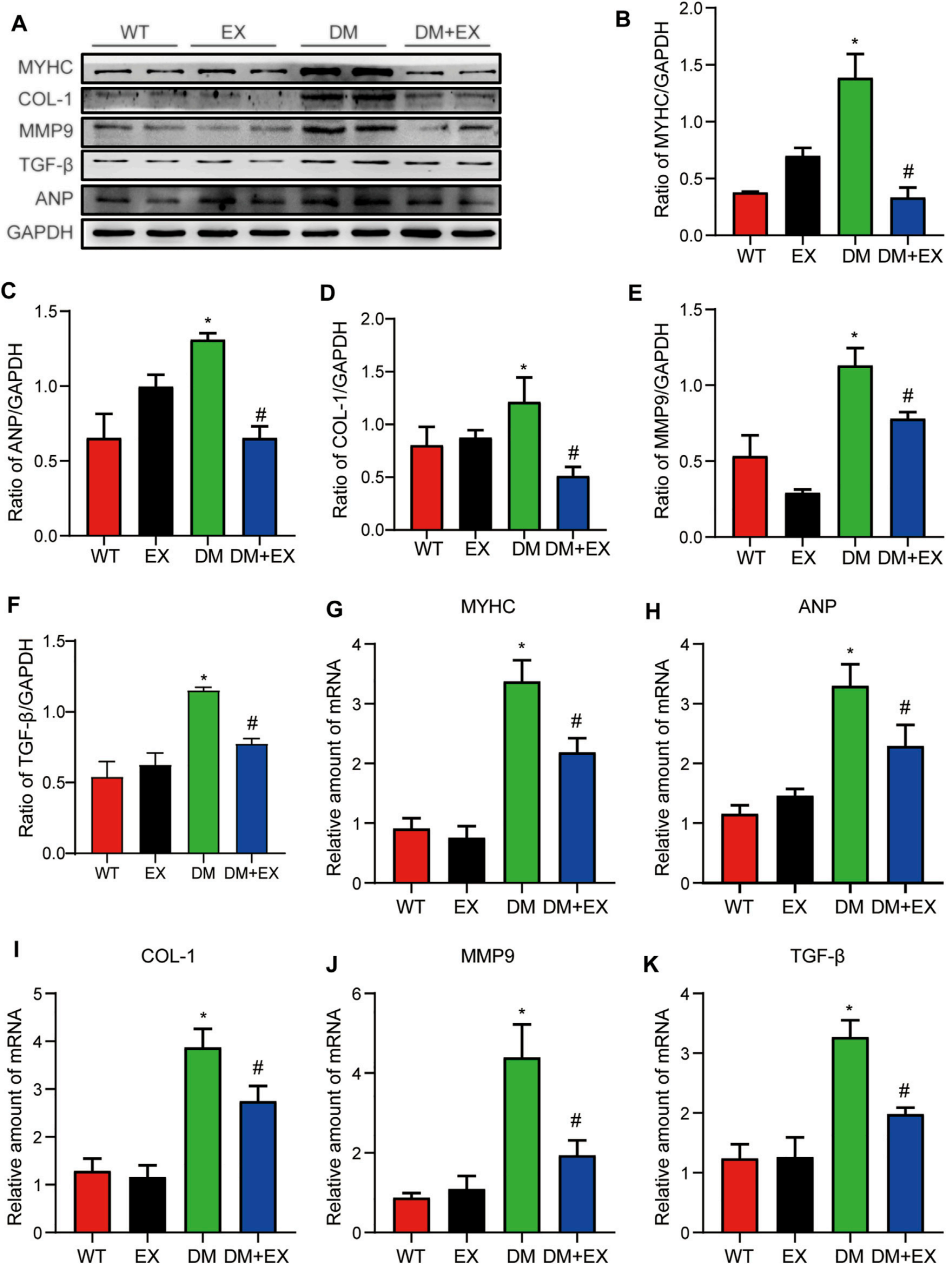
Exercise weakened myocardial remodeling in diabetic mice. **(A–F)** Protein expression of fibrotic markers and hypertrophy in myocardial tissue and Statistical Graph. **(G–K)** Gene expression of myocardial hypertrophic markers and fibrosis in myocardial tissue. *p < 0.05 vs. the WT group, ^#^ *p* < 0.05 vs. the db/db group.

**FIGURE 8:**
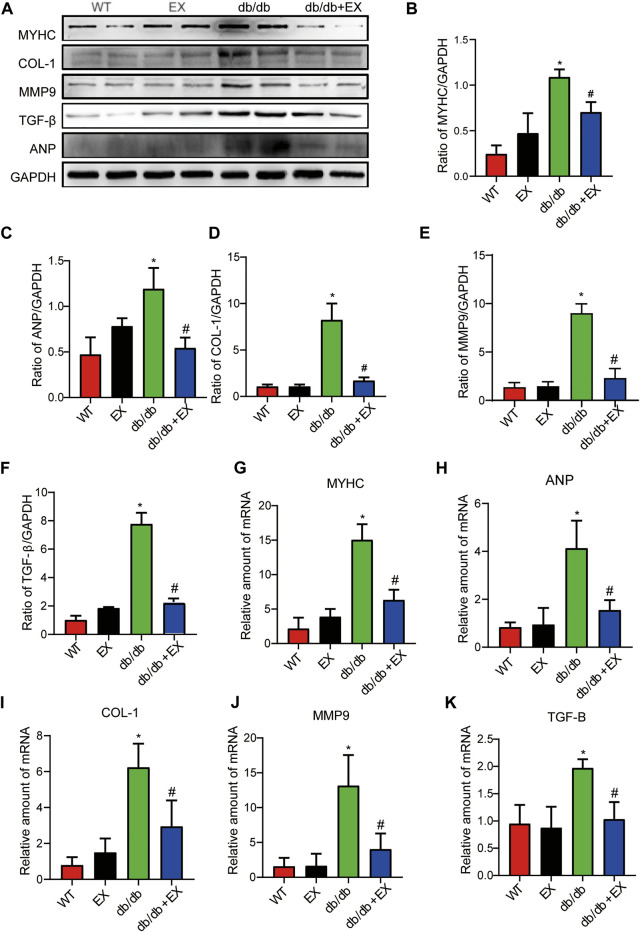
Aerobic exercise ameliorated myocardial remodeling in DM mice. **(A–K)** Changes in protein and mRNA levels of hypertrophic fibrosis indicators. *p < 0.05 vs. the WT group, ^#^ *p* < 0.05 vs. the DM group.

In addition, fibrosis is an important pathological variation in DCM. The connective cardiac tissue was determined by Sirius Red staining for collagen. Compared with WT mice, the hearts from the db/db group and DM group showed apparent collagen and fibrous tissue accumulation, which were reduced by regular exercise training (Figure 6). As for the protein level, the profibrotic makers, including TGF-β, COL-I and MMP9, and cardiac hypertrophic markers, including MyHC and ANP, were significantly elevated in the heart tissues of the db/db group or DM group. Moreover, the results of PCR for these genes level showed the same tendency. These molecular biological changes were remarkably inhibited by aerobic exercise training (Figure 7, 8). Overall, these experimental results suggested that aerobic exercise significantly improved myocardial remodeling in diabetic mice.

### Exercise Training and P2X7R Deficiency Ameliorated Cardiac Remodeling in HFD + STZ-Induced Type 2 Diabetes Mellitus Mice

Considering that P2X7R expression was upregulated in the db/db group or DM group and significantly decreased after exercise, we hypothesized that P2X7R may be involved in the pathophysiological process of exercise in improving DCM in mice (Figure 1). To further investigate the role of P2X7R in the exercise model of diabetic mice, P2X7R knockout mice were conducted and treated with HFD and STZ injection to induce the T2DM model. No significant difference was found in body weight and blood glucose level between the P2X7R^−/−^ + DM group and C57BL/6 background DM mice, as well as between the P2X7R^−/−^ + EX group and EX group (Table 3), indicating that P2X7R knockout had no effect on blood glucose level.

**TABLE 3:** Effects of exercise on the biochemistry of diabetic mice (±SD, *n* = 6).

	DM	DM + EX	P2X7R^−/−^ + DM	P2X7R^−/−^ + DM+EX	P2X7R^−/−^	P2X7R^−/−^ + EX
BW, g	27.05 ± 01.51	29.84 ± 1.93	23.11 ± 1.89	27.61 ± 3.23	28.87 ± 1.32	33.04 ± 3.26
HW, mg	142.9 ± 17.06	135.5 ± 18.69	130.8 ± 9.2	5.10 ± 0.43	156.1 ± 7.13	171.0 ± 20.28
HW/BW, mg/g	5.10 ± 0.43	5.04 ± 0.49	5.05 ± 0.32	4.73 ± 0.38	5.41 ± 0.11	5.37 ± 0.32
Glucose, mmol/L	26.46 ± 4.8	29.11 ± 3.99	25.7 ± 8.66	25.06 ± 7.35	10.57 ± 1.35	10.83 ± 1.30
IVSd, mm	0.69 ± 0.04	0.70 ± 0.03	0.63 ± 0.05*	0.62 ± 0.09^#^	0.75 ± 0.03	0.75 ± 0.05
LVIDd,mm	4.3 ± 0.22	4.117 ± 0.209	4.058 ± 0.11	3.927 ± 0.20	4.096 ± 0.17	4.204 ± 0.15
LVPWd, mm	0.69 ± 0.049	0.6989 ± 0.04122	0.61 ± 0.06*	0.6307 ± 0.09^#^	0.7236 ± 0.2111	0.7333 ± 0.06
LVIDs, mm	2.79 ± 0.38	2.678 ± 0.201	2.561 ± 0.43	2.65 ± 0.89	2.694 ± 0.2468	2.617 ± 0.29
EF%	58.83 ± 1.17	69.5 ± 2.929*	67.71 ± 3.77*	75.13 ± 3.23^#&^	76.83 ± 3.19	75.67 ± 5.66
FS%	29 ± 2.83	33.54 ± 2.03*	33.8 ± 2.17*	38.4 ± 2.41^#^	39.75 ± 2.63	41 ± 2.31

Echocardiographic assessment of cardiac function parameters of each group of experimental mice. BW, body weight; HW, heart weight; HW/BW, heart weight/body weight; IVSd, diastolic interventricular septal thickness; LVIDd, diastolic left ventricle internal volume; LVPWd, left ventricular posterior wall thickness; LVIDs, left ventricular end-systolic diameter; EF%, ejection fraction; FS%, fraction shortening (*n* = 6; *p < 0.05 vs. the DM group, ^#^ *p* < 0.05 vs. the DM + EX group, ^&^ *p* < 0.05 vs. the P2X7^−/−^ + DM group).

In addition, all mice were tested for echocardiography 2 h before sacrifice to evaluate cardiac function. As shown in Table 3, under basal conditions, the P2X7R^−/−^ group had no effect on cardiac function compared with the WT group (Table 3). Moreover, systolic dysfunction (as shown in EF% and FS% indices) was observed in the DM group, which improved by P2X7R deficiency.

**FIGURE 9:**
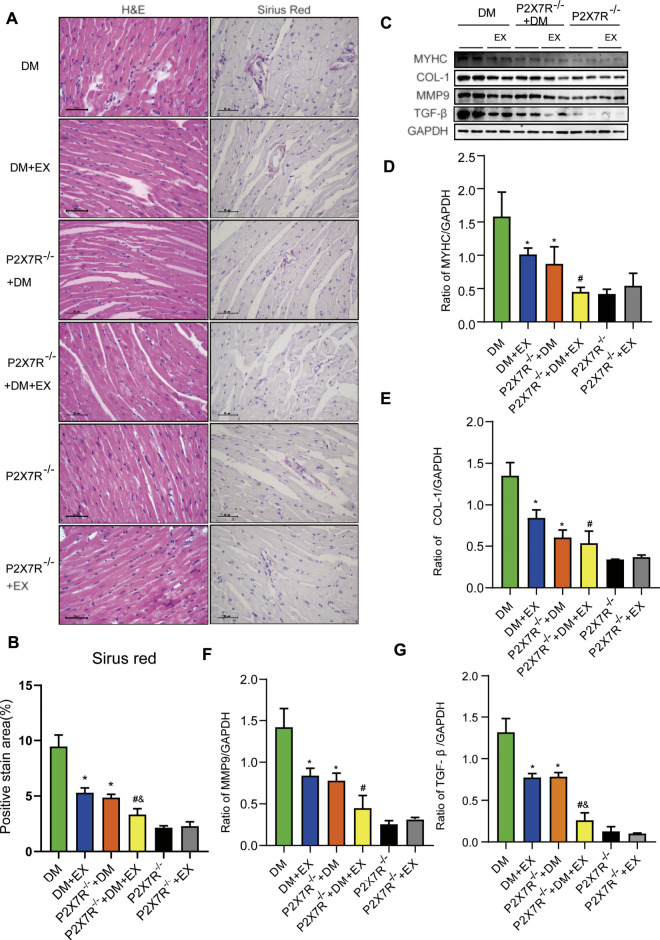
Myocardial remodeling and collagen deposition in the T2DM model of P2X7R knockout mice induced by HFD and STZ injection. **(A,B)** Hematoxylin–eosin (H&E) and Sirius red staining quantitative statistical images of Sirius red detecting myocardial fibrosis. **(C–G)** Proteins levels of fibrotic markers (e.g., COL-1, TGF-β, and MMP9) and hypertrophic markers (e.g., MyHC) in myocardial tissues were measured using Western blotting. *p < 0.05 vs. the DM group, ^#^ *p* < 0.05 vs. the DM + EX group.

**FIGURE 10:**
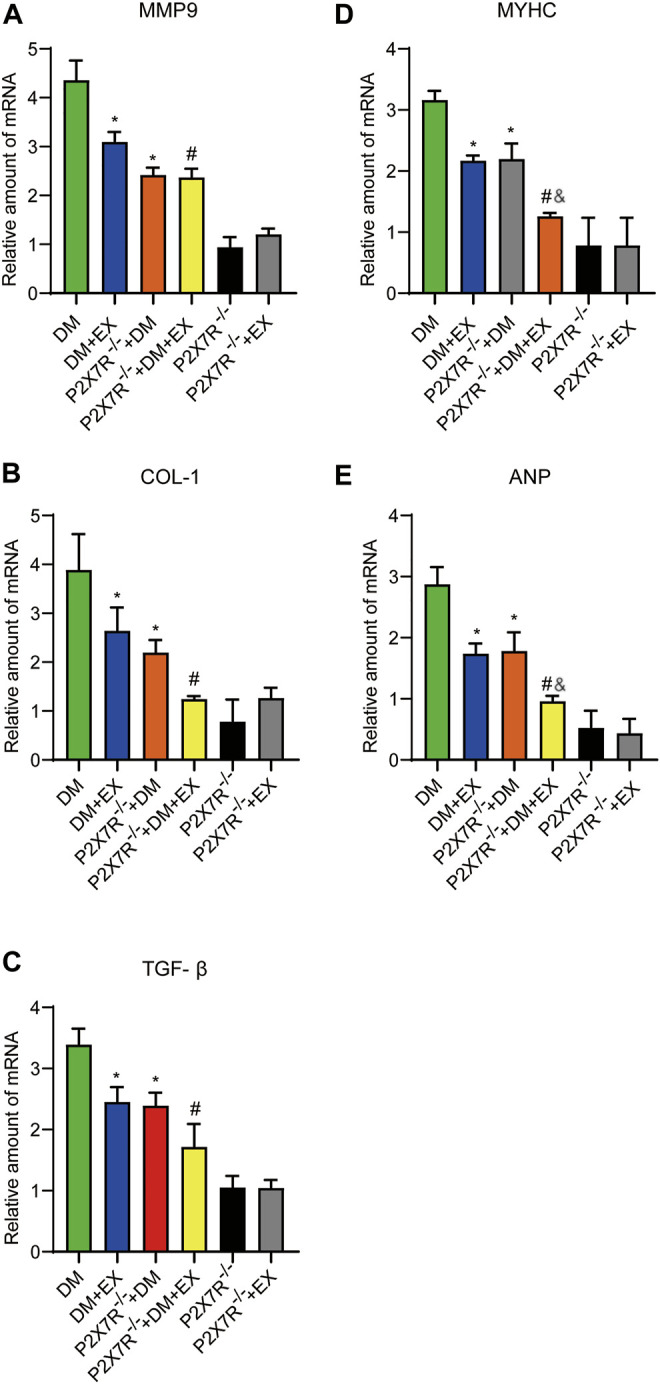
The statistics of Real-time polymerase chain reaction (PCR) for fibrosis, hypertrophy and apoptosis indicators. The levels of fibrotic markers (e.g., COL-1, TGF-β, and MMP9) and hypertrophic markers (e.g., MyHC and ANP) **(A–E)**. *p < 0.05 vs. the DM group, ^#^ *p* < 0.05 vs. the DM + EX group. ^&^ *p* < 0.05 vs. the P2X7R^−/−^ + DM group.

**FIGURE 11:**
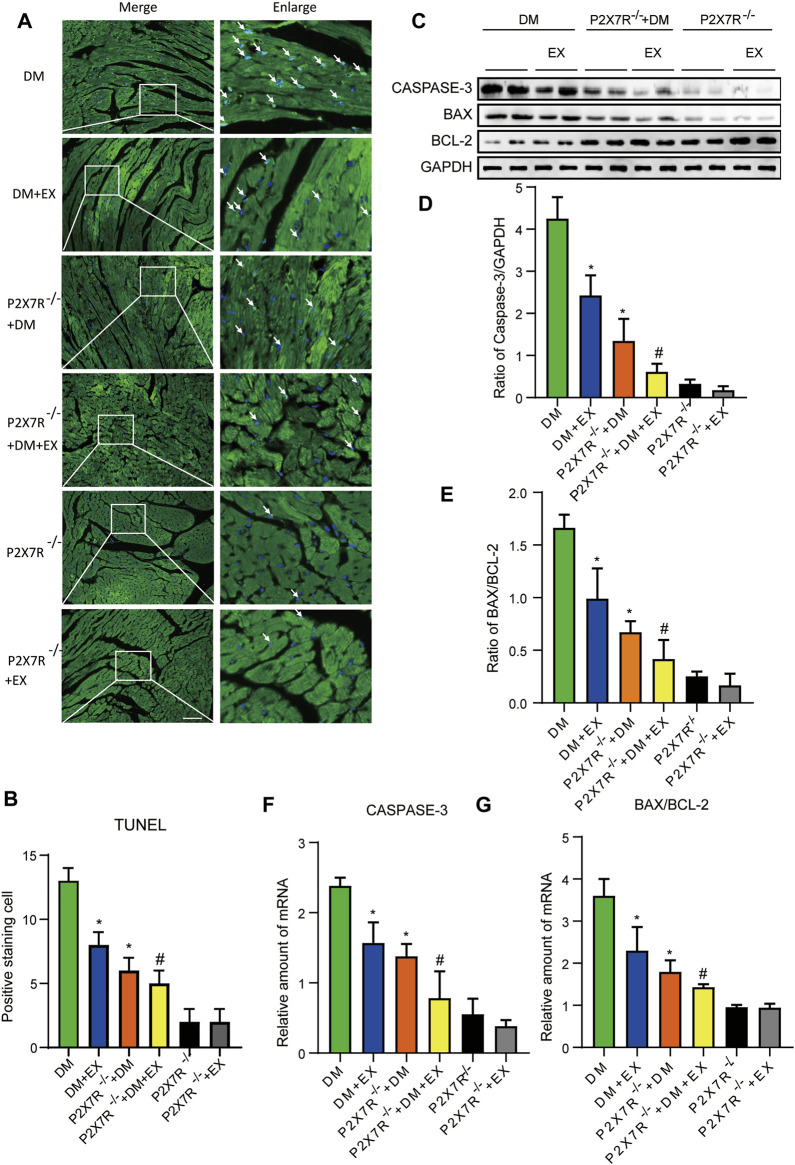
P2X7R knockout or exercise training had positive effects on reducing myocardial apoptosis. **(A,B)** The images and quantitative statistics of TUNEL staining for apoptosis of myocardial cells in diabetic mice. **(C–G)** Statistical map of protein and gene expression of apoptosis-related protein (Caspase-3, Bax, and Bcl-2) in myocardial tissues were measured using Western blotting and PCR. *p < 0.05 vs. the DM group, ^#^ *p* < 0.05 vs. the DM + EX group.

With regard to changes in myocardial structure, apparent structural abnormalities, such as disorderly arranged muscle fiber, were observed in the HFD + STZ-induced DM group and typically reversed by P2X7R knock out or aerobic exercise treatment (Figures 9A,B). In addition, the hearts of diabetic mice exhibited evident deposition of fibrous tissue and collagen tissue. Similarly, the expression of profibrotic indicators (TGF-β, COL-1, and MMP9) and cardiac hypertrophic markers (MyHC and ANP) were evidently increased in diabetic mice. Interestingly, these pathophysiological changes were reversed by the DM + EX group or P2X7R^−/−^ + DM group (Figures 9, 10). These results suggested that exercise or the deficiency of P2X7R can effectively improve myocardial fibrosis and hypertrophy. Next, we further assessed the levels of apoptosis in cardiac tissues. Based on the data obtained from TUNEL staining, P2X7R knockout or exercise training had positive effects on reducing myocardial apoptosis and protecting the survival of cardiomyocytes in diabetic mice (Figures 11A,B). Moreover, HFD + STZ treatment increased Caspase 3 and Bax expression and inhibited the expression of Bcl-2 in myocardial tissue. And as we expected, these abnormal changes were decreased in the P2X7R^−/−^ + DM group or DM + EX group (Figures 11C–G).

Considering that aerobic exercise typically reduced P2X7R expression and ameliorated cardiac remodeling in diabetic mice, we evaluated the effect of P2X7R knockout combined with exercise treatment on T2DM mice. Strikingly, in the P2X7R^−/−^ + DM + EX group, we observed evident improvement in cardiac dysfunction, dramatically alleviated cardiac fibrosis and hypertrophy, and decreased myocardial apoptosis level compared with the DM + EX group (Figures 10,11). Compared with the P2X7R^−/−^ + DM group, the P2X7R^−/−^ + DM + EX group represented a lower level of cardiac fibrosis (Figures 9A,B). The expression of TGF-β at the protein level and the level of TGF-β and ANP at the genetic level evidently decreased in the P2X7R^−/−^ + DM + EX group, while it did not significantly differ from other indicators (Figures 9, 10). These results indicated that exercise can effectively alleviate myocardial apoptosis and alleviate myocardial fibrosis at least in part by inhibiting P2X7R.

### Exercise Training or P2X7R Deficiency Inhibited LncRNA MIAT, Increased miR-150 in Diabetic Mice

**FIGURE 12:**
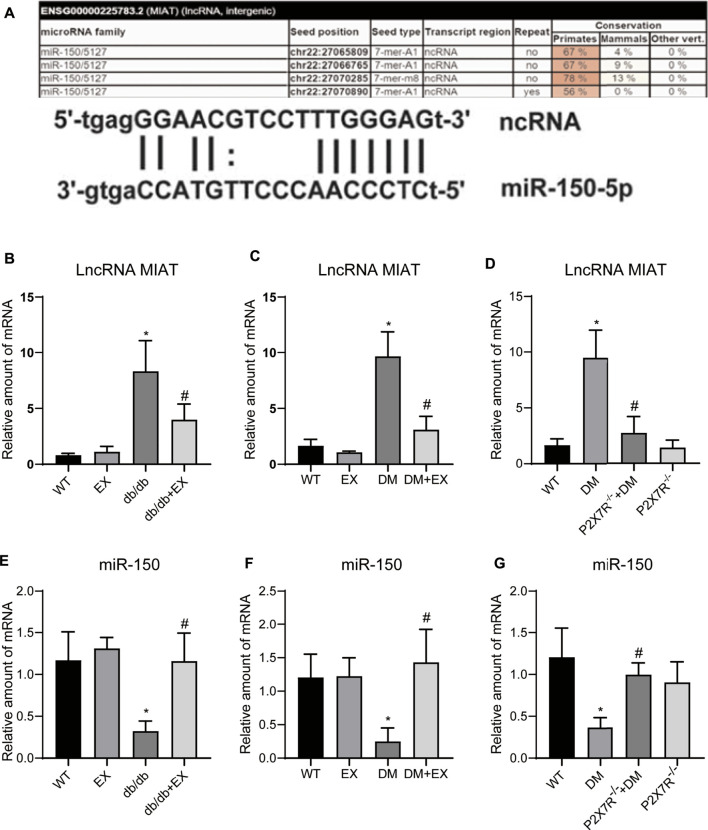
P2X7R knockout and exercise decreased lncRNA MIAT and increased miR-150. **(A)**The binding site of lncRNA MIAT and miR-150. **(B–G)** The level of lncRNA MIAT and miR-150 in db/db mice and the DM group. *p < 0.05 vs. the WT group, ^#^ *p* < 0.05 vs. db/db group or the DM group.

Given that lncRNA MIAT and miR-150 have base binding sites (Figure 12A), and was responsible for the cardiac hypertrophy and fibrosis (Qu et al., 2017; Yang et al., 2021), we investigated the expression levels of LncRNA MIAT and miR-150 (Figures 12B–G). We found that lncRNA MIAT was significantly increased and miR-150 expression was down-regulated in cardiac tissues of diabetic mice. Notably, exercise training can markedly inhibit the expression of lncRNA MIAT and up-regulate miR-150. Also, in the P2X7^−/−^ + DM group, we found that compared with the DM group, the expression of lncRNA MIAT was decreased, while miR-150 was increased, suggesting lncRNA MIAT/miR-150 may be the downstream mechanism of P2X7R for exercise training to regulate myocardial remodeling in diabetic mice.

## Discussion

In this study, we revealed the role of aerobic exercise on a mouse model of T2DM with or without P2X7R deficiency. We found that P2X7R expression significantly increased in db/db mice and HFD + STZ-induced DM mice, accompanied by cardiac dysfunction and enhancement of myocardial fibrosis and apoptosis. Aerobic exercise significantly inhibited P2X7R expression, reduced myocardial apoptosis and relieved cardiac fibrosis and hypertrophy. In addition, depletion of P2X7R effectively reversed these abnormal changes. We showed that P2X7R^−/−^ + DM + EX displayed more evidently improvement in cardiac remodeling, dysfunction, and apoptosis in T2DM mice compared with the DM + EX group, suggesting the beneficial effect of exercise training on DCM could partly inhibit P2X7R. Collectively, our data revealed the pivotal role of P2X7R in the pathophysiology of T2DM, and aerobic exercise can improve myocardial remodeling, at least partly via P2X7R-dependent mechanisms in DCM.

Myocardial remodeling is an important feature of many cardiovascular diseases, which primarily appears as interstitial, perivascular fibrosis and hypertrophy and impaired heart function. In DCM, apparent cardiac abnormality lies different mechanisms, namely, inflammation, mitochondrial dysfunction, and apoptosis, which ultimately lead to the extracellular cardiac remodeling and eventually heart failure (Palomer et al., 2018; Zhou et al., 2020). Reversing these detrimental processes will improve the cardiac function of patients with diabetes mellitus. Here, we observed consistent pathological changes of cardiac tissues from a mouse model of db/db mice or HFD + STZ-induced T2DM mice. Interestingly, in both models of T2DM mice, we found that P2X7R was abundantly expressed in cardiac tissues. In addition, the depletion of P2X7R effectively alleviated cardiac remodeling and improved cardiac systolic dysfunction in T2DM mice. This result was consistent with our previous study in the model of T1DM (Huang et al., 2021), which indicates P2X7R deficiency ameliorates cardiac injury and remodeling in mice by PKCβ and ERK. Collectively, P2X7R, as a potential target for the therapeutic management, plays an important role in the regulation of heart function in DCM.

Mechanically, among the pathological mechanisms of DCM that have been reported (Parim et al., 2019), apoptosis of cardiomyocytes is the main cause of DCM progression. In this study, we confirmed that the expression of apoptosis-related proteins and the number of apoptosis-positive cells in TUNEL staining were increased in db/db mice or HFD + STZ-treated T2DM mice and bluntly decreased in the exercise group.

For decades, regular exercise has been found to delay or prevent some complications caused by diabetes, although it induces physiological cardiac hypertrophy through cardiac cell growth (Mcgavock et al., 2004; Seo et al., 2019). A growing body of evidence indicates that aerobic exercise can improve DCM. In brief, regular aerobic exercise presents significant improvements in cardiac function and remodeling, improved glucose and insulin metabolism (Zheng et al., 2018; Verboven et al., 2019), and decreases cardiac fibrosis, apoptosis, and oxidative stress, thereby reducing the risk factors for cardiovascular disease (Seo et al., 2019). In addition, clinical significance of exercise for DCM is observed (Taylor et al., 2014; Da Silva et al., 2019), and over the long term, 150 min or more of moderate-to-vigorous intensity aerobic activity every week is recommended to be the protective strategy against the development of DCM according to the 2019 American Diabetes Association guidelines (American Diabetes Association, 2019). Consistent with the above-mentioned results, here we elucidated that exercise can reagainst DCM, much of literature in this field indicates that exercise can regulate cardiomyocyte metabolism by increasing GLUT-4 expression or reducinduce myocardial cell apoptosis and myocardial fibrosis in diabetic mice and improve cardiac dysfunction in T2DM. Thus, exercise plays an essential role in the regulation of cardiac function.

For the potential protective mechanism roles of exercise g Forkhead box protein O1 (Röhling et al., 2016) and play a role in the regulation of calcium, mitochondrial function, oxidative stress, and cardiac ultrastructural changes (Searls et al., 2004; Hafstad et al., 2015). In the present study, we revealed that increased P2X7R expression caused by diabetes was inhibited by regular exercise, and the depletion of P2X7R effectively reversed cardiac remodeling, indicating that exercise improves cardiac dysfunction caused by diabetes by regulating P2X7R. These data provide supportive evidence which P2X7R is another potential target involved in the effect of exercise on DCM. Notably, we next further underscored this effect in HFD + STZ-induced mice by combining P2X7R knockout with exercise treatment. More prominent beneficial effect on the heart was observed in the P2X7R^−/−^ + DM + EX group compared with the DM + EX group. Moreover, compared with the P2X7R^−/−^ + DM group, the P2X7R^−/−^ + DM + EX group reduced myocardial collagen deposition, which indicated that exercise alleviated cardiac remodeling in DCM partly by inhibiting P2X7R expression.

Previous studies have shown that long non-coding RNAs (lncRNAs) are associated with the pathological development of cardiac hypertrophy. For example, lncRNA H19 (Liu et al., 2016) and lncRNA-ROR (Jiang et al., 2016) promote cardiac hypertrophy. And in Ang II-induced mouse myocardial hypertrophy model and H9c2 cells, lncRNA MIAT overexpression can promote hypertrophy by sponging miR-150 (Zhu et al., 2016), and which upregulate in diabetic hearts and promote cardiac fibrosis (Jin, 2021; Aonuma et al., 2022). These evidences indicate a link effect of lncRNA MIAT/miR-150 contributes to cardiac remodeling. Interestingly, we indicated that LncRNA MIRT was significantly increased and miR-150 was greatly decreased in the cardiac tissues of mice with type 2 diabetes mellitus. However, exercise training or P2X7R knockout mice reversed the changes. Taken these data, and considering some base binding sites exists between lncRNA MIAT and miR-150, we speculated lncRNA MIRT/miR-150 may be downstream mechanism for exercise training to inhibit P2X7R improving myocardial remodeling in diabetic mice (Figure 12). Additionally, Liu Yang et al. showed that ablation of lncRNA MIAT attenuates pathological hypertrophy and heart failure in both angiotensin II infusion and transverse aortic constriction model (Yang et al., 2021), and alleviated cardiac fibrosis. Considering the result from rats with mycardial infarction, the positive effect of exercise training in attenuating cardiac fibrosis partially mediated through reducing of lncRNA MIAT (Farsangi et al., 2021), it seems that lncRNA MIAT may be a potential therapeutic target in the regulation of various cardiac remodeling. However, more detailed investigations are needed to explore the exact molecular link between exercise and lncRNA MIAT, and confirm the exact relationship between lncRNA MIAT and miR-150.

## Conclusion

This study systematically revealed the role of exercise in reducing P2X7R expression, modulating cardiac fibrosis and apoptosis, and improving cardiac dysfunction in DCM at least partly by inhibiting the P2X7R levels of cardiac tissues. Therefore, exercise training, as an effective non-pharmacologic measure made out of a tailored exercise prescription would positively affect DCM management in the future.

## Data Availability

The original contributions presented in the study are included in the article/Supplementary Materials, further inquiries can be directed to the corresponding authors.

## Glossary

Bax: Bcl-2 associated X protein
Bcl-2: B cell lymphoma/lewkmia-2
BW: weight
CAD: coronary artery disease
Caspase-3: cysteinyl aspartate specific proteinase-3
CHOL: total cholesterol
CK: creatine kinase
COL-1: collagen I
db/db: db/db mice
db/db + EX: db/db mice plus exercise
DCM: diabetic cardiomyopathy
DM + EX: HFD + STZ induced T2DM mice plus exercise
DM: HFD + STZ induced T2DM mice
EF: ejection fraction
EX: excised
FS: fractional shortening
GAPDH: glyceraldehyde-phosphate dehydrogenase
H&E: Hematoxylin and Eosin
HFD: high-fat diet
HW: heart weight
HW/BW: heart weight/body weight
IVSd: diastolic interventricular septal thickness
lncRNAs: long non-coding RNAs
LVIDd: diastolic left ventricle internal volume;
LVIDs: left ventricular end-systolic diameter
LVPWd: left ventricular posterior wall thickness
MMP9: matrix metalloproteinase-9
MyHC: myosin heavy chain
P2X7R: P2X7 purinergic receptors
**P2X7R** ^**−/−**^: Purinergic P2X7 receptor knockout mice
**P2X7R** ^**−/−**^ **+ DM**: Purinergic P2X7 receptor knockout and HFD + STZ induced T2DM mice
**P2X7R** ^**−/−**^ **+ EX**: Purinergic P2X7 receptor knockout mice plus exercise
**P2X7R** ^**−/−**^ **+ DM + EX**: Purinergic P2X7 receptor knockout and HFD + STZ induced T2DM mice plus exercise
PCR: real-time polymerase chain reaction
RNA: ribonucleic acid
ROS: reactive oxygen species
STZ: streptozotin
T2DM: type 2 diabetes mellitus
TGF-β: fibrotic growth factor beta
TRIG: triglycerides
TUNEL: terminal deoxynucleotidyl transferase-mediated DUTP nick end labeling
WT: the control group
